# Anti-GD2 Directed Immunotherapy for High-Risk and Metastatic Neuroblastoma

**DOI:** 10.3390/biom12030358

**Published:** 2022-02-24

**Authors:** Godfrey Chi-Fung Chan, Carol Matias Chan

**Affiliations:** 1 Department of Pediatrics & Adolescent Medicine, The University of Hong Kong, Pokfulam, Hong Kong SAR, China; 2 Department of Pediatrics, Hong Kong Children’s Hospital, Kowloon Bay, Hong Kong SAR, China; 3 Department of Pediatrics, Hong Kong University-Shenzhen Hospital, Shenzhen 518058, China; 4 Faculty of Medicine, University of Central Lancashire, Preston PR1 7BH, UK; cmchan2@uclan.ac.uk

**Keywords:** anti-GD2, neuroblastoma, immunotherapy

## Abstract

Neuroblastoma is one of the few childhood cancers that carries a tumor-specific antigen in the form of a glycolipid antigen known as GD2. It has restricted expression in normal tissue, such as peripheral afferent nerves. Monoclonal antibodies targeting GD2 have been applied clinically to high-risk neuroblastoma with significant success. However, there are different anti-GD2 products and administration regimens. For example, anti-GD2 has been used in combination with chemotherapy during the induction phase or with retinoic acid during the maintenance stage. Regimens also vary in the choice of whether to add cytokines (i.e., IL-2, GMCSF, or both). Furthermore, the addition of an immune enhancer, such as β-glucan, or allogeneic natural killer cells also becomes a confounder in the interpretation. The question concerning which product or method of administration is superior remains to be determined. So far, most studies agree that adding anti-GD2 to the conventional treatment protocol can achieve better short- to intermediate-term event-free and overall survival, but the long-term efficacy remains to be verified. How to improve its efficacy is another challenge. Late relapse and central nervous system metastasis have emerged as new problems. The methods to overcome the mechanisms related to immune evasion or resistance to immunotherapy represent new challenges to be resolved. The newer anti-GD2 strategies, such as bispecific antibody linking of anti-GD2 with activated T cells or chimeric antigen receptor T cells, are currently under clinical trials, and they may become promising alternatives. The use of anti-GD2/GD3 tumor vaccine is a novel and potential approach to minimizing late relapse. How to induce GD2 expression from tumor cells using the epigenetic approach is a hot topic nowadays. We expect that anti-GD2 treatment can serve as a model for the use of monoclonal antibody immunotherapy against cancers in the future.

## 1. Introduction

Immunotherapy is a novel emerging anti-cancer strategy in recent years. There are different forms of immunotherapy, and they can be categorized into groups based on their mechanism of action: (1) cytokines, such as interferon, inducing host immune response [1]; (2) monoclonal antibodies, such as anti-CD20 [2], anti-EGFR [3], anti-VEGF [4], and anti-GD2 [5], targeting tumor-specific antigens; (3) immune cellular therapy, including cytokine-induced killer (CIK) cells [6], dendritic cells (DCs) [7], natural killer (NK) cells [8], and chimeric antigen receptor T (CAR-T) cells [9]; (4) immune checkpoint inhibitors, including programmed cell death 1 (PD-1), programmed cell death ligand 1 (PDL-1), and cytotoxic T lymphocyte-associated protein 4 (CTLA-4) inhibitors [10]; and (5) bioengineered oncolytic viruses [11] or bacteria [12]. These strategies can be applied simultaneously, such as combining cytokines (e.g., IL-2) with monoclonal antibodies or immune checkpoint inhibitors with immune cellular therapy. To date, the most widely used immune therapies are monoclonal antibodies, immune cellular therapy (especially allogeneic hematopoietic stem cell transplant (HSCT)), and immune checkpoint inhibitors.

In pediatric solid tumors, due to the identification of relatively few tumor-specific antigens, the application of immunotherapy is lagging that of adult counterparts [13]. However, there are exceptions, and one of them is neuroblastoma. Neuroblastoma is a common malignant solid tumor in childhood, and high-risk or metastatic disease is found in more than 50% of cases, with a poor prognosis [14]. Over the past two decades, the discovery of monoclonal antibodies against neuroblastoma-specific surface antigens known as gangliosides [15,16] and their subsequent application have improved outcomes in high-risk neuroblastoma significantly [5,17,18].

**Figure 1:**
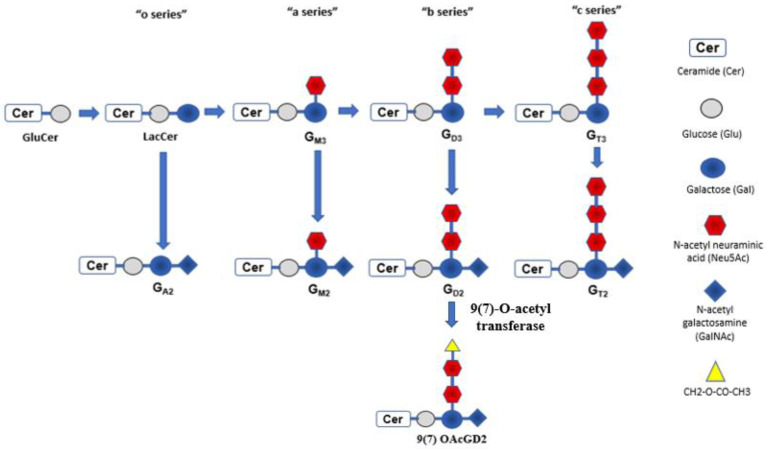
Gangliosides can be classified into four series: 0-, a-, b-, and c-series. They differ based on the number of N-acetylneuraminic acids (Neu5Acs) involved in sialic acid chain. GD2 and GD3 have two Neu5Acs and differ by the presence of N-acetyl galactosamine (GalNAc) or not. GD2 has GalNAc and adding *O*-acetyl group to N-acetyl Neu5Ac will form the subgroup of GD2 antigen known as O-acetylated GD2 (OAcGD2). It can be targeted by monoclonal antibody 8B6mAb. LacCer, lactosylceramide.

Almost all neuroblastoma cells express glycolipid antigens known as gangliosides. Gangliosides are sialic acid-containing glycosphingolipids that can be classified into four series: 0-, a-, b-, and c-series (Figure 1) [19]. They differ from each other based on the number of N-acetylneuraminic acids involved in the sialic acid chain. Gangliosides are mainly engaged in signal transduction, cell adhesion, and recognition. Normal tissues usually express a-series gangliosides. Neuroblastoma expresses b-series gangliosides rather than a-series, as aberrant glycosylation is a hallmark of malignant cellular transformation. This includes disialoganglioside (GD2) and hematoside (GD3). As expected, b-series gangliosides have a restricted expression pattern in normal tissues. For example, GD2 expresses during fetal development, then gradually fades, and is subsequently found in mature peripheral afferent nerves and skin melanocytes.

Other than neuroblastoma, many neuroectoderm-derived tumors and sarcomas also express GD2 [20], including a wide variety of childhood cancers such as osteosarcoma, retinoblastoma, melanoma, brain tumors (e.g., diffuse intrinsic pontine glioma), rhabdomyosarcoma, and Ewing sarcoma. It is also expressed in some adult cancers, including small-cell lung and breast carcinomas [21]. However, the expression level of GD2 in these tumors differs, with neuroblastoma showing the highest expression level. It is known that GD2 enhances the adhesion and invasion of neuroblastoma cells, so it facilitates the metastatic process [22].

**Figure 2:**
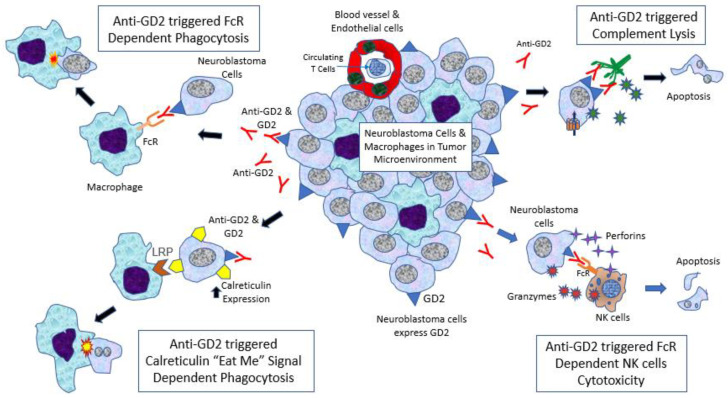
Neuroblastoma tumor microenvironment and cytotoxic action induced by anti-GD2. (1) Anti-GD2 can trigger complementary activation by C1q–antibody interaction, leading to complement lysis of neuroblastoma cells (complement dependent cytotoxicity (CDC)). (2) Anti-GD2 activates natural killer (NK) cells via FcγRIIIA (CD16a), leading to release of perforins and granzymes that can kill neuroblastoma cells (antibody-dependent cellular cytotoxicity (ADCC)). (3) Anti-GD2 can activate macrophages via FcγRI (CD64) and FcγRIIA (CD32), leading to initiation of phagocytosis of neuroblastoma cells (antibody-dependent phagocytosis (ADP)). (4) Monoclonal antibodies such as anti-GD2 can induce calreticulin (“eat me” molecule) expression on neuroblastoma cells and enhance phagocytosis by macrophages.

The ways in which anti-GD2 exerts its anti-cancer function are summarized in Figure 2. In pediatric solid tumors, including neuroblastoma, they are infiltrated by macrophages rather than lymphocytes [23]. Anti-GD2 can trigger complementary activation by the C1q–antibody interaction, leading to complement lysis of neuroblastoma cells [24,25]. In fact, this is also the postulated mechanism of pain induced by anti-GD2, which is due to complement lysis of GD2-expressed afferent neurons. Another mechanism is antibody-dependent cellular cytotoxicity (ADCC), which involves natural killer (NK) cell activation via Fc receptors, mainly FcγRIIIA (CD16a) [26,27]. Fc receptors of NK cells interact with the Fc fragment of anti-GD2, then trigger the release of toxic molecules, perforins and granzymes, leading to lysis of the targeted cells. This is considered by most experts as the key anti-neuroblastoma mechanism of anti-GD2. Interleukin-2 (IL-2) was given with anti-GD2 in Children’s Oncology Group (COG) and International Society of Pediatric Oncology European Neuroblastoma Group (SIOPEN) protocols to enhance NK cell proliferation [28]. Finally, another important anti-GD2 mechanism is antibody-dependent phagocytosis. Monoclonal antibodies such as anti-GD2 can activate macrophages via various Fc receptors, particularly FcγRI (CD64) and FcγRIIA (CD32) [29], then facilitate phagocytosis of neuroblastoma cells. FcγRIIA (R/R) polymorphism in macrophages is associated with better progression-free survival in patients receiving anti-GD2 with GM-CSF [30]. However, the latest research suggests that this interaction alone may not be potent enough,; the cells should also express an interesting “eat me” molecule, known as calreticulin [31]. It was found that anti-GD2, but not other monoclonal antibodies such as anti-B7H3, can induce calreticulin expression on neuroblastoma cells and enhance phagocytosis by macrophages (Majzner, SIOP 2021).

## 2. Development of Anti-GD2 Antibody for Therapeutic Use

Identification of GD2 and GD3 expression on neuroblastoma cells can be dated back to the 1980s [32,33], and shortly thereafter, the first generation of anti-GD2 antibody of murine origin (3F8) was developed [34]. Due to the possibility of inducing human anti-mouse antibody (HAMA) [35], this form of monoclonal antibody was subsequently replaced by chimeric antibody. This improved version merges the variable region of the murine immunoglobulin into the constant region (Fc) of the human immunoglobulin backbone. Commercial chimeric anti-GD2 consists of Ch14.18/SP2.0 (dinutuximab, Unituxin^®^, United Therapeutics; COG) [36,37], Ch14.18/CHO (dinutuximab beta, Qarziba^®^, EUSA; SIOPEN) [38], and Ch14.18K332A (Provenance Biopharmaceuticals; St. Jude) [39]. Recently, further refinement of the bioengineering process led to the incorporation of the complementary segment of murine immunoglobulin to the human immunoglobulin structure (humanized antibody). Humanized anti-GD2 consists of hu14.18-IL2 (NCI; COG) [40] and hu3F8 (naxitamab, Danyelza^®^, Y-mAbs; MSKCC) [41]. However, some patients still develop neutralizing antibodies against either the chimeric or humanized antibody, known as a human anti-chimeric antibody (HACA) [42] or human anti-humanized antibody (HAHA) [41].

Whether HAMA, HACA, and HAHA have any clinical relevance in terms of their impact on anti-GD2 efficacy remains to be verified [43]. In fact, it was shown that anti-GD2 may generate a cascade of secondary (Ab2) and tertiary (Ab3) anti-idiotypic antibodies in vivo. Interestingly, Ab3 also possesses anti-tumor properties [44,45]. Based on this observation, a study correlating patients with or without HAMA and anti-anti-idiotype antibodies (Ab3) with outcome supports this hypothesis [46,47]. Consequently, phase I and II tumor vaccine trials aimed at inducing GD2 anti-idiotypic antibody formation are ongoing, with encouraging results [48,49,50].

## 3. Different Anti-GD2 Preparations and Their Pros and Cons

Currently, there are no clinical data to determine which anti-GD2 preparation is superior to the others, but there are in vitro data that can give us a glimpse of their differences. It has been shown that hu3F8 has a 10-fold higher affinity to the GD2 target than Ch14.18 [51] but with a shorter half-life. In terms of cytotoxic potency, Ch14.18K332A has greater antibody-dependent cellular cytotoxicity (ADCC) by both NK cells and neutrophils [52]. However, its bioengineered design to avoid complement lysis-induced pain means that it does not have complement-dependent cellular cytotoxicity, whereas Ch14.18 appears to have a lower affinity and cytotoxic potency but a longer half-life. Whether all of these characteristics have an impact on the treatment outcome remains to be tested in the future [52].

Naxitamab is a newer form of humanized (IgG1) anti-GD2 (hu3F8) monoclonal antibody. It was initially developed by Cheung at Memorial Sloan Kettering Cancer Center. Y-mAbs Therapeutics Inc., which was formed by a group of patients’ parents, then acquired the commercial license for naxitamab for the treatment of neuroblastoma and osteosarcoma. Naxitamab with granulocyte-macrophage colony-stimulating factor was recently given accelerated approval by the US FDA for the treatment of pediatric patients >1 year of age with mainly relapsed or refractory high-risk neuroblastoma [53].

For the two Ch14.18 monoclonal antibodies, the production cell lines being used affects the post-translational modification (PTM) process. Non-human mammalian cell lines such as CHO and SP2/0 produce PTMs that are not expressed in humans, *N*-glycans known as galactose-α1,3-galactose (α-Gal) and *N*-glycolylneuraminic acid [54]. As most human subjects have acquired circulating antibodies against these N-glycans, they can induce an anaphylactic reaction [55]. It was shown that the CHO cell line generates much less α-Gal than the SP2/0 cell line. As a result, based on the published data [17,56], the incidence of grade 3 or 4 allergic reaction to dinutuximab was 25%, but only 10% for dinutuximab beta. The incidence of anaphylactic reaction was 18% for dinutuximab and only 0.8% for dinutuximab beta.

Anti-GD2 treatment is associated with significant neuropathic pain for almost all patients. Ch14.18K332A is a chimeric anti-GD2 with the complement binding domain removed [57]. It is believed that the complement lysis triggered by anti-GD2 on the afferent nerve induces an intense pain sensation. Taking away the complement binding site can potentially minimize the anodynia. It was presumed that the anti-tumor efficacy would not be compromised, as the main mechanism of anti-tumor action is through ADCC by natural killer cells.

Another approach to minimize the pain is to target a subgroup of GD2 antigen known as O-acetylated GD2 (OAcGD2) by monoclonal antibody 8B6mAb [58]. The study found that GD2 could further metabolize into 9(7) OAcGD2 ganglioside by 9(7)-O-acetyl transferase (Figure 1). OAcGD2 is poorly immunogenic and found to be overexpressed in both pediatric and adult solid tumors cells [59]. It is also in cancer stem cells, but has little or no expression in normal tissue [60]. Since OAcGD2 does not express in normal cells, including neurons, the pain is theoretically minimized. However, such specific change in target also affects the possible mode of action of 8B6mAb, the antibody-dependent phagocytosis (ADP) exerted by macrophages is the main cytotoxic mechanism involved by 8B6. Such cytotoxic effect may be hindered by the upregulation of CD47 expression on neuroblastoma cells in vivo [61]. CD47 is also known as the “don’t eat me” molecule against the phagocytic action of macrophage. Antibody specifically blocking the CD47 receptor of macrophage, known as SIRPα, can restore antibody-dependent phagocytosis toward NB cells and re-establish the anti-neuroblastoma activity [62]. Such an approach could also be extrapolated to other forms of anti-GD2 therapy in the future.

In addition to these preparations, anti-GD2 has been tagged with radioactive molecules for either diagnostic or therapeutic purposes [63]. Immunocytokines are a new class of molecules synthesized by bioengineering techniques to link specific tumor monoclonal antibodies with activating cytokines. For example, anti-GD2 can link to an IL-2 molecule to enhance the cytotoxicity [64,65].

## 4. Clinical Data on Anti-GD2 for Newly Diagnosed Patients

Among the registered clinical trials involving the use of anti-GD2, nine completed the studies. Excluding those pharmacokinetic studies and small phase I trials, we explored the respective anti-GD2 administration regimens and results. The details, including dosage and road map, are presented in specific figures. The results are summarized in Table 1. One important thing to note is the recruitment criteria. Many studies included “high-risk”, patients which meant even early-stage or stage 4S patients with *MYCN* amplification or infants (<18 months) with metastatic disease not meeting the criteria of stage 4S. Those patients have a better prognosis, which could impact the estimation of survival.

**Figure 3:**
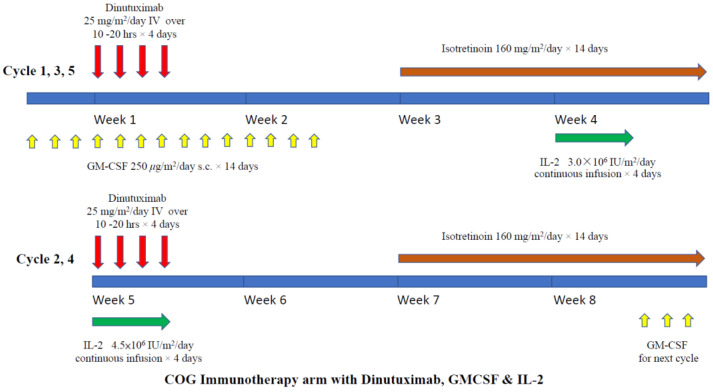
Treatment schema of COG maintenance immunotherapy (dinutuximab) with GM-CSF and IL-2.

A randomized phase III trial was conducted by the Children Oncology Group (COG), showing that anti-GD2 ch14.18 (dinutuximab) plus GM-CSF and IL-2 could enhance the survival of children with high-risk neuroblastoma [17]. The study included metastatic neuroblastoma patients (including those <18 months except stage 4S patients) and high-risk patients, such as those with localized disease with *MYCN* amplification. All children received standard chemotherapy, surgery, local irradiation, and autologous peripheral blood stem cell transplantation (auto-PBSCT). They were then randomized to receive maintenance treatment with either isotretinoin alone or isotretinoin with dinutuximab. Patients in the dinutuximab arm received the drug for four consecutive days, in four-weekly cycles for five cycles. In cycles one, three, and five, daily GM-CSF was given, and in cycles two and four, IL-2 was added. The detailed treatment scheme is shown in Figure 3. The results showed that dinutuximab was superior to isotretinoin alone in both 2 years event-free survival (EFS; 66% vs. 46%, *p* = 0.01) and overall survival (OS; 86% vs. 75%, *p* = 0.02). Based on this result, dinutuximab was approved by the FDA [66]. Subsequent long-term follow-up of the same cohort confirmed that both 5 years EFS and OS remained superior compared to the control arm (EFS: 57% vs. 46%, *p* = 0.042 and OS: 73% vs. 57%, *p* = 0.045) (Table 1) [43]. However, late relapses were observed in patients in the dinutuximab arm and did not reach the plateau. What additional strategy can help to prevent late relapse is one of the challenges now. The extended study confirmed that the outcome and toxicity profile have no correlation with the plasma level of dinutuximab, HACA, or sIL2R.

Another multi-center clinical trial using anti-GD2 was the German NB97 study. In this non-randomized cohort study, patients with stage 4 neuroblastoma (>1 year) received six cycles of ch14.18 as maintenance therapy [67]. Another 69 patients, who did not receive Ch14.18 due to either refusal or other reasons, were recruited as controls. No additional cytokines were used. Nine-year event-free survival (EFS) and overall survival (OS) were 41 and 46%, respectively (Table 1). The OS, but not the EFS, of the anti-GD2 arm was better than that of the control arm (*p* = 0.019). This suggests that the use of anti-GD2 alone during the maintenance phase without enhancement with cytokines may not generate an optimal immune response.

**Figure 4:**
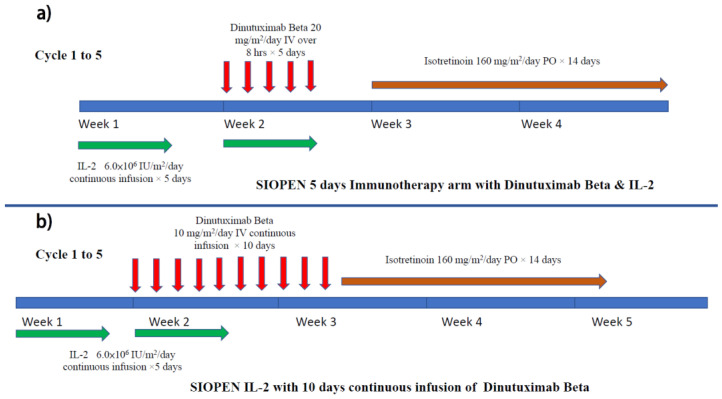
Treatment schema of (**a**) SIOPEN maintenance immunotherapy (dinutuximab beta) with IL-2 and (**b**) SIOPEN continuous infusion immunotherapy with IL-2 for relapsed neuroblastoma.

The SIOPEN group subsequently conducted a multi-center phase 3 randomized clinical trial in which ch14.18/CHO (dinutuximab beta) was given as maintenance therapy with or without concomitant use of subcutaneous IL-2 [56]. Eligible patients completed the multidrug induction regimen (Rapid COJEC or N7) then underwent high-dose therapy followed by auto-PBSCT rescue. Focal radiotherapy targeted at the primary tumor site was performed after the transplant. For maintenance treatment, patients were randomized to either dinutuximab beta alone or dinutuximab beta with IL-2 (Figure 4a). Dinutuximab beta was given as an 8 hrs infusion for five days. In the combined treatment arm, high-dose IL-2 (double that used in the COG trial) was given. The 3 years EFS for dinutuximab beta alone vs. dinutuximab beta with subcutaneous IL-2 was not statistically significant (Table 1). Hypersensitivity was the most common grade 3–4 adverse event; the rate was 10% in the dinutuximab beta alone group, but double that in the IL-2 group (20%). The study concluded that adding IL-2 to dinutuximab beta did not improve outcomes of patients with high-risk neuroblastoma but was associated with more toxicity. There were also comments that the negative benefit of IL-2 in this study was due to the high IL-2 dosage [68].

The same group also analyzed their immunotherapy cohort (2009 to 2013, *n* = 378) with the historical control group, who received standard treatment alone (2002 to 2009, *n* = 466) [18]. It was found that the anti-GD2 cohort had significantly better EFS and OS (Table 1). Multivariate analysis showed that no immunotherapy, incomplete response prior to anti-GD2 treatment, and the involvement of more than one metastatic compartment at diagnosis were significant risk factors for relapse or disease progression.

Around the same time as the COG and German NB97 studies, another study using anti-GD2 (murine 3F8) also published its results. Children with stage 4 neuroblastoma after achieving complete remission (CR) or good partial remission (*n* = 169), with intensive chemotherapy with or without autologous HSCT, received murine 3F8 monoclonal antibody plus GMCSF [5]. They were stratified into three groups: (1) group A (*n* = 43) received murine 3F8 alone, (2) group B (*n* = 41) received 3F8 plus intravenous GMCSF, and (3) group C (*n* = 57) received 3F8 with subcutaneous GMCSF. In addition, another 28 patients belonging to the ultra-high-risk category also received the group C regimen. The 5 years PFS was 44% (95% CI, 32–62), 56% (95% CI, 43–74), and 62% (95% CI, 50–76) for regimens A, B, and C, respectively (*p* = 0.018). The 5 years OS showed a similar trend of 49% (95% CI, 36–66), 61% (95% CI, 48–78), and 81% (95% CI, 70–92) for regimens A, B, C respectively (*p* = 0.003). For the ultra-high-risk group, 5 years PFS and OS were 36% (95% CI, 22–59) and 75% (95% CI, 60–93), respectively, which is very impressive for this group of patients. This cohort study suggests that anti-GD2 with subcutaneous GMCSF apparently yields better results. Another observation was that if patients failed to achieve remission, as defined by molecular MRD detection (with markers for cyclin D1, PHOX2B, and GD2 synthase) after two courses of anti-GD2 treatment, they would have poorer PFS and OS, with a hazard ratio of 6.6 and 7.9, respectively. However, this result must be confirmed by a randomized clinical trial.

**Figure 5:**
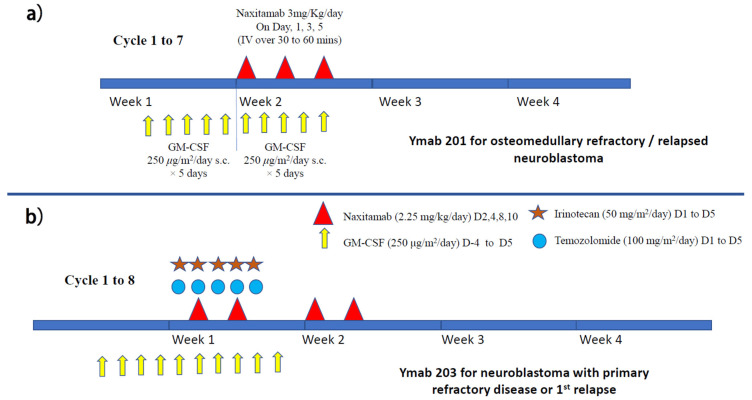
Treatment schema of (**a**) naxitamab maintenance immunotherapy with GM-CSF (for patients with CR1 (protocol 202) or osteomedullary refractory disease/relapse (protocol 201)) and (**b**) naxitamab immunotherapy with GM-CSF for neuroblastoma patients with fist relapse associated with soft tissue lesion (protocol 203).

**Figure 6:**
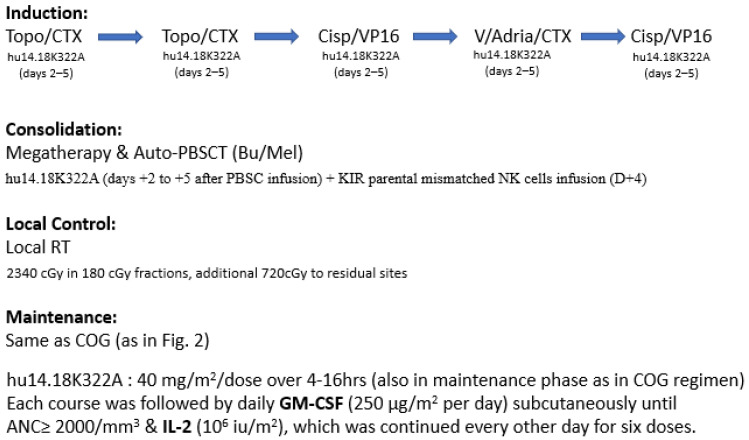
Treatment schema of St. Jude immunotherapy (hu14.18K322A) with GM-CSF and IL-2 during induction and maintenance phases. Some patients also received haploidentical NK cell infusion after auto-PBSCT.

Murine 3F8 was later upgraded to humanized 3F8 (naxitamab), and several international clinical trials (protocols 201and 203) are ongoing (Figure 5a,b). A single-center study using naxitamab for high-risk neuroblastoma patients at their first or second CR has been reported. Seventy-three high-risk neuroblastoma patients (stage M at age > 18 months or MYCN-amplified stage L1/L2 at any age) were given N7-based chemotherapy regimens followed by naxitamab and subcutaneous GM-CSF. Treatment consisted of five cycles of GM-CSF for five days, followed by naxitamab with a double dose of GM-CSF for another five days (Figure 6a). Naxitamab was given as a 30 min infusion on days one, three, and five in an outpatient setting. Fifty-eight patients (79.5%) completed the therapy. Three-year EFS and OS were 58.4 +/− 14.9%, and 82.4 +/− 15.8% for the whole cohort. Four patients (5%) developed grade 4 toxicity and 10 patients (14%) suffered from early relapse. The 3 years EFS and OS for patients with first CR were 74.3 +/− 13.8% and 91.6 +/− 8.4%, respectively. Significant differences in EFS can be found between patients with first and second CR (*p* = 0.0029). The pattern of relapse was mainly isolated organ (75%), mostly bone (54%) [69]. How to achieve a better remission rate is the challenge. The follow-up period of this study was relatively short, and upcoming international trials will verify the result.

While most of the anti-GD2 clinical trials applied the immunotherapy as consolidation during the maintenance phase, the St. Jude group added hu14.18K322A to the induction chemotherapy period in a single-arm phase II clinical trial [70]. Six courses of hu14.18K322A were given together with induction chemotherapy, followed by GM-CSF and low-dose IL-2 (Figure 6). Megadose chemotherapy followed by auto-PBSCT was performed with busulfan and melphalan conditioning. After transplant, an additional course of hu14.18K322A was given with parental-derived natural killer cells in patients when killer immunoglobulin receptor (KIR) mismatched parental donor was available. As consolidation, the conventional COG maintenance regimen of anti-GD2 with GM-CSF, IL-2, and isotretinoin was adopted. This regimen was well tolerated, and impressively, no patients experienced treatment failure with disease progression during induction. This may have an impact on long-term outcomes, since failure to achieve remission is one of the main factors in treatment failure, as shown in previous clinical trials. In an update report with more patients recruited (*n* = 64), still no patient developed progressive disease after induction. The 3 years EFS was 73.7% (95% CI, 60–83) and the OS was 86% (95% CI, 74–93) [71]. COG and SIOPEN are both currently designing trials to verify this approach.

So far, no study directly compared the clinical efficacy of different forms of anti-GD2. We have experience in using murine 3F8 (n = 68), dinutuximab beta (*n* = 36), and naxitamab (*n* = 8, mainly relapse or refractory cases). For our newly diagnosed patients receiving maintenance immunotherapy with either murine 3F8 (without GMCSF or IL-2) or dinutuximab beta (with or without IL-2), five-year EFS and OS were comparable to the reported results. As in the long-term COG study, we also noticed late relapse (longest at 8.5 years) in our long-term follow-up cohort (mainly with murine 3F8).

## 5. Clinical Data on Anti-GD2 for Relapsed Patients

Whether anti-GD2 has any therapeutic effect for patients with relapsed or refractory neuroblastoma was proven by a randomized trial conducted by the COG (ANBL1221) [72]. The trial aimed to compare the effect of dinutuximab (17.5–25 mg/m^2^/day on days two–five) versus temsirolimus (mTOR inhibitor, 35 mg/m^2^/day on day one and eight). Both were used together with chemotherapy in the form of irinotecan (50 mg/m^2^/day for five days) and temozolomide (100 mg/m^2^/day for five days) on a 21-day cycle. GMCSF was given subcutaneously on days 6 to 12 for those who received dinutuximab. Thirty-five patients were recruited (18 in the temsirolimus arm and 17 in the dinutuximab arm). The study was closed prematurely before the targeted enrolment was attained (medium follow-up 1.26 years), since the response rate was far better in the dinutuximab arm (53%; 95% CI 29.2–76.7) than the temsirolimus arm (6%; 95% CI 0.0–16.1). The most common side effect that could be attributed to dinutuximab was pain (44%).

To reduce pain, continuous long-term infusion of dinutuximab beta was given over 10 days for five to six cycles (35 days per cycle) together with high-dose subcutaneous IL-2 (days 1–5, 8–12) to a group of patients with relapsed neuroblastoma (Figure 5b). It was found that this prolonged infusion method did not affect the immunogenicity of ch14.18/CHO and ADCC. On the other hand, the pain reaction was markedly reduced by prolonging the infusion time. To facilitate the administration, patients could be discharged with a mobile infusion pump after the first cycle (Lode, SIOPEN progress report). Despite previous exposure to anti-GD2, most patients still showed a significant response to the anti-GD2 treatment [73].

A phase 1 single-center clinical trial was performed on children >1 year of age with resistant or recurrent neuroblastoma [74]. Fifty-seven patients received three doses of naxitamab alone via short-term intravenous infusion (over 30 mins) on alternate days, preceded by subcutaneous GMCSF starting five days before naxitamab infusion (Figure 5a). The treatment was given in an outpatient setting. This was a dose-finding study, and no maximum tolerated dose was identified, even when the dosage was increased to around 2.5 times higher than the conventional dosage. The main side effects were pain and hypotension. A substantial number of patients showed significant response. Under the same treatment regimen, another 27 patients with relapsed/refractory bone or bone marrow disease were treated with naxitamab alone. Biopsies were performed on persistent MIBG avid lesions but showed non-restrictive patterns on MRI (apparent diffusion coefficient >1) and/or low or negative ^18^FDG-PET uptake (SUVmax < 2). Interestingly, histology showed that 10/16 specimens (62.5%) differentiated into fully mature neuroblasts [75]. This suggests that persistent MIBG lesions with a negative PET signal may not require further treatment. A phase 3 international multi-center study on patients with relapsed/refractory bone or bone marrow disease (Y-mAbs 201) is currently ongoing to verify the efficacy of this approach.

The incidence of neuroblastoma metastasizing to the central nervous system was reported to increase, while the survival of neuroblastoma patients improved [76]. It was estimated to account for around 3% (53/1977) of relapsed neuroblastoma patients based on SIOPEN pooled data [77]. Patients with *MYCN* amplification or metastatic involvement at more than one site (especially liver) are particularly prone to this adverse event. The inability of anti-GD2 monoclonal antibodies to pass through the blood–brain barrier may be one of the contributory factors. It appears that the incidence of central nervous system relapse remains the same for patients who received high-dose therapy either with or without immunotherapy [77].

For patients with central nervous system (CNS) relapse, intraventricular radioimmunotherapy using I^131^-3F8 or I^131^-8H9 (targeting B7H3, omburtamab, Y-mAbs) may help to clear the neuroblastoma cells in the cerebrospinal fluid [78]. Out of the 21 patients treated, 17 patients remained alive without CNS disease at a median follow-up of 33 months (range 7–74 months). For the four dead cases, only one patient has evidence of residual CNS disease. The incidence of radionecrosis using intraventricular radioimmunotherapy either alone or with conventional external beam craniospinal irradiation is low (around 1%). In long-term follow-up, no significant neurologic deficits related to radionecrosis were observed in this cohort. The outcome with radioimmunotherapy was quite impressive; up to 65% of patients survived for five years [79].

## 6. Side Effects of Anti-GD2

Since anti-GD2 is often given concomitantly with cytokines, including GMCSF or/and IL-2, the side effects may be related to the additive effects of these agents. As shown in Table 2, the side effects directly related to anti-GD2 are reflected by the dinutuximab without IL-2 treatment data from the SIOPEN trial. Other than pain and fever, it appears that myelosuppression is also common with dinutuximab beta, and the addition of IL-2 further aggravates this side effect. It is known that mesenchymal stem cells (MSCs) in the bone marrow also expresses GD2 [80], and MSCs serves as a niche for hematopoiesis [81]. Whether anti-GD2 treatment partly affects the marrow compartment requires further investigation.

Anti-GD2-induced allodynia basically occurs in most patients; it was initially described as “delayed extreme pain syndrome” in 5 of 12 melanoma patients receiving murine anti-GD2 [82]. Subsequently, allodynia was found to be the most common adverse effect encountered [83]. The pain can present as abdominal discomfort, fleeting pain over the limbs, and headache. Gabapentin, a gamma-aminobutyric acid analogue, may reduce this form of neuropathic pain [84], while during the infusion, morphine is often needed to reduce the pain. A study found that prolonging the infusion time, such as administering a continuous infusion over 10 days, can reduce the incidence and intensity of anti-GD2 induced allodynia [73]. The intensity of pain can be markedly increased with a short infusion regimen (i.e., 30 min), and morphine alone may not be effective under such approach. Based on our experience and that of Mora et al., ketamine at a dose of 1 to 2 mg/kg can effectively control the pain if morphine fails.

Though it was reported as uncommon, mydriasis and impaired accommodation were found in a group of children with either refractory or relapsed neuroblastoma receiving anti-GD2 antibody [85]. In patients who received escalating doses of hu14.18K322A, ranging from 2 to 70 mg/m^2^/dose for 4 consecutive days in a 28-day cycle, mydriasis was identified in 13/38 patients (34%), and loss of accommodation to light was seen in 9 (24%). It was postulated that this may be dose related. In fact, this complication has been found with both Ch14.18 and 3F8 related anti-GD2 [86]. We found this complication in 1/36 (2.8%) and 2/8 (25%) of our patients on dinutuximab beta and naxitamab, respectively. It can be missed if it is not actively searched for. Patients with mydriasis can wear tinted spectacles to minimize the photophobia. Ocular symptoms resolved in most patients after the drug was stopped, therefore patients with this complication should continue with anti-GD2 treatment.

Rarely, more severe demyelinating polyneuropathy can occur after anti-GD2 treatment [87], and several cases have been reported after Ch14.18 and Ch14.18/CHO treatment. Most of the patients eventually recovered, but some patients seemed to have long-lasting or permanent disability (SIOPEN Annual Workshop 2020).

## 7. Future Prospective

Currently, there are several ongoing clinical trials involving the use of various forms of anti-GD2, and they have all yielded reasonably good results [88]. However, despite the use of anti-GD2, almost 50% of patients still relapsed. Understanding the underlying resistant mechanism is important in order to find an appropriate measure to circumvent the obstacles. Many immune-evasive mechanisms have been reported [89].

Indoleamine-pyrrole 2,3-dioxygenase1 (IDO1), which converts tryptophan into kynurenine, has been implicated in the mechanism of neuroblastoma immunotolerance to immune cells. IDO1 can block IFN-γ production of both NK cells and T cells and hence to reduce their cytotoxicity [90]. Therefore, blocking the IDO1-related pathway may enhance the response to immunotherapy. Currently, there is a dual IDO1/TDO inhibitor (RY103), which has been demonstrated to be effective in suppressing IDO1 in a pre-clinical murine pancreatic cancer model [91].

Another challenge is the low number of immune cells, including cytotoxic T cells (CD8+) and NK cells (CD56+), in the tumor microenvironment [23]. This will undermine the effect of antibody treatment. To circumvent the low quantity and function of T cells in children with cancer, a new approach is to use bispecific antibodies linking hu3F8 to T cells. This has been shown to have potent antitumor cytotoxicity against GD2(+) tumors in vitro and in vivo [92]. T cells can be expanded in vitro and then attached to hu3F8-BsAb for clinical application. This can improve the efficacy of monoclonal antibodies.

Another approach is to use an epigenetic agent to alter the activity profile of immune cells. Pre-clinical animal data suggest that there is a synergy between the HDAC inhibitor vorinostat and anti-GD2 mAbs [93]. In mice with adrenal tumors treated with vorinostat, many more infiltrative myeloid cells and macrophages were found in the tumor microenvironment. These innate immune cells were shown to have increased MHC-II and Fc-receptor expression, suggesting a more active immune reaction against cancer cells after epigenetic manipulation.

Recently, a pilot trial of a GD2/GD3 vaccine showed promising results. The vaccine was given as seven subcutaneous injections over one year. In addition, oral beta-glucan was given as an immune enhancer after the third vaccine dose. Up to 32% of patients achieved progression-free survival. The IgG1 titer and a specific dectin-1 SNP, rs3901533, were associated with better survival [50]. Dectin-1 is a known beta-glucan receptor, and beta-glucan can enhance dendritic cell maturation [94,95].

Another immune therapy against GD2 is GD2-directed chimeric antigen receptor T cells (CAR-T) cells. However, based on the initial experience of using CAR-T for solid tumors, it is not as promising as in hemic malignancies [96,97]. Another concern is the potential neurotoxicity, as suggested by an animal study [98]. While anti-GD2 monoclonal antibody cannot pass through the blood–brain barrier, CAR-T cells are not bound by such restriction. In addition, CAR-T cells can potentially be maintained within the recipient’s body for a long time. The other challenge is the diverse immune-suppressive mechanisms exerted by the cancer microenvironment. How to overcome such adversity remains to be solved [99].

In summary, at present, at least three anti-GD2 monoclonal antibodies have been approved by health authorities as acceptable treatment options for high-risk and metastatic neuroblastoma. Clinical trials have shown that the three products yielded similar results, which is much better compared to chemotherapy alone. However, a significant proportion of patients still fail with this approach. How to enhance the efficacy of immunotherapy requires further research to overcome the immune-resistant mechanisms. Combinations of bispecific antibodies with either autologous or allogeneic T cells, or immune checkpoint inhibitors with CAR-T cells, are currently being tested clinically, and the results will guide us toward better utilization of immunotherapy for neuroblastoma in the future.

## Figures and Tables

**Table 1:** Results of multi-center trials comparing anti-GD2 containing regimens with conventional treatments.

References	Immunotherapy Included	Treatment Schedule	Number of Subjects	Median Follow-Up	EFS	OS
Yu A, et al., 2011	Dinutuximab, GMCSF, IL-2	As maintenance after chemotherapy and auto-PBSCT	Randomized trial N = 113 (anti-GD2) N = 113 (standard)	2.1 years (4 days–6.9 years)	2 years EFS 66 ± 5% (anti-GD2) 46 ± 5% (standard)	2 years OS 86 ± 4% (anti-GD2) 75 ± 5% (standard)
Yu A, et al., 2021 (follow-up study)	Same as above	Same as above	Same as above	9.97 years (0.7 years–5.3 years)	5 years EFS 56.6 ± 5% (anti-GD2) 46.1 ± 5% (standard)	5 years OS 73.2 ± 4% (anti-GD2) 56.6 ± 5% (standard)
Simon T, et al., 2011	Ch14.18 (BioInvent)	As maintenance after chemotherapy with or without auto-PBSCT	Non-randomized cohort study N = 164 (anti-GD2)	11.1 years (2.3 years–8.6 years)	5 years EFS 51.3 ± 6% (anti-GD2) 34.1 ± 5% (standard) 9 years EFS 44.7 ± 6% (anti-GD2) 31 ± 5% (standard)	5 years OS 60.3 ± 6% (anti-GD2) 42.2 ± 5% (standard) 9 years EFS 46.6 ± 6% (anti-GD2) 33.9 ± 5% (standard)
Ladenstein R, et al., 2018	Dinutuximab β, with or without IL-2	As maintenance after chemotherapy and auto-PBSCT	Randomized trial N = 206 (anti-GD2 + Il2) N = 200 (anti-GD2)	4.7 years (3.9 years–5.3 years)	5 years EFS 53 ± 7% (anti-GD2) 60 ± 6% (anti-GD2 + IL-2)	5 years OS 63 ± 8% (anti-GD2) 62 ± 7% (anti-GD2 + IL-2)
Ladenstein R, et al., 2020	Dinutuximab β, with or without IL-2	As maintenance after chemotherapy and auto-PBSCT	Non-randomized cohort study (Historical control) N = 378 (anti-GD2) N = 466 (standard)	5.8 years (4.2 years–8.2 years) 4.6 years for anti-GD2 & 8.6 years for standard arm	5 years EFS 57 ± 6% (anti-GD2) 42 ± 5% (standard)	5 years EFS 64 ± 5% (anti-GD2) 50 ± 5% (standard)

**Table 2:** Overview of side effects of three commercially available anti-GD2 products based on their respective clinical trials, modified to match symptoms for comparison.

Adverse Events (Gr 3 or 4)	hu3F8 (+GMCSF) (Mora et al.)	Dinutuximab β (no IL-2) (Ladenstein, et al.)	Dinutuximab β (+IL-2) (Ladenstein, et al.)	Dinutuximab (+IL-2 & GMCSF) (Yu, et al.)
Hypotension	63%	7%	17%	18%
Pain	65%	66%	86%	52%
Urtricaria	29%	5%	10%	13%
Pyrexia	2%	14%	40%	39%
Bronchospasm	21%	0%	0%	0%
Elevated ALT/AST	0%	17%	23%	23%
Nausea & vomiting/diarrhea	2%/0%	5%/7%	9%/21%	6%/13%
Deranged renal function	0%	2%	1%	Hypokalemia (35%) Hyponatremia (23%)
Neutropenia	15%	33%	58%	-
Anemia	0%	42%	66%	-
Lethargy	10%	0%	6%	0%
Hypoxia	10%	0%	0%	13%
Allergy	0%	10%	20%	25%
Neuropathy	0%	3%	9%	4%

## Data Availability

The data were extracted from published literature in opened access domain.
